# Supplementary material for: Differential lateral and basal tension drive folding of Drosophila wing discs through two distinct mechanisms
Source: Nat Commun. 2018 Nov 5;9:4620. doi: 10.1038/s41467-018-06497-3 (PMC6218478; doi:10.1038/s41467-018-06497-3)
Supplement: Supplementary file 1 — Supplementary Information [file 41467_2018_6497_MOESM1_ESM.pdf]

Supplementary Information

Differential lateral and basal tension drive folding of *Drosophila* wing discs through two distinct mechanisms

Sui, et al.

Supplementary Figures

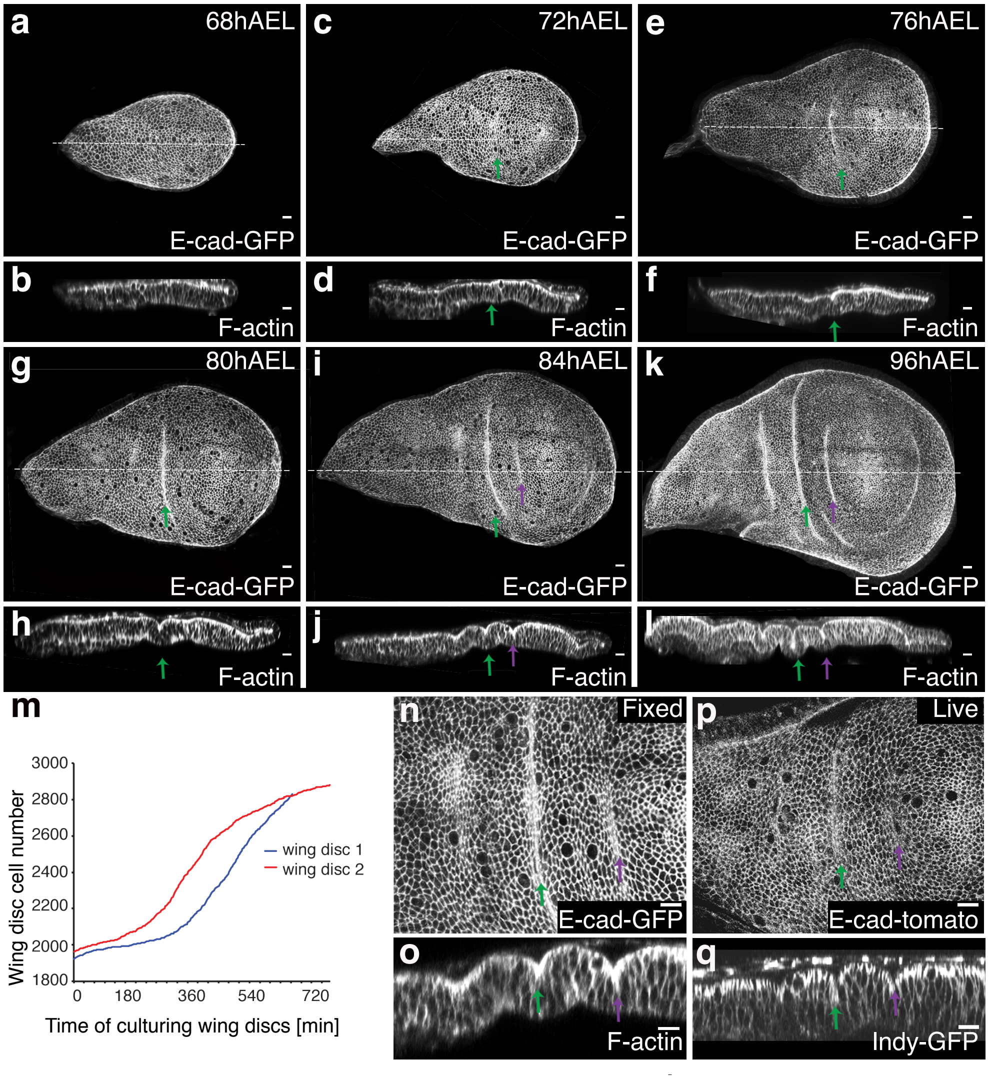

Supplementary Figure 1. Analysis of fold formation in fixed and live wing discs

(a-l) Top view (a,c,e,g,i,k) and cross-sectional images (b,d,f,h,j,l) of fixed wing discs of larvae of the indicated times after egg lay (AEL) stained for E-cadherin-GFP and F-actin. Scale bars are 10 μm. H/H fold (green arrow) and H/P fold (magenta arrow) are indicated.

(m) Number of cells in a wing disc as a function of time in culture is shown.

(n-q) Top view (n,p) and cross-sectional images (o,q) of a fixed wing disc of 84h AEL larva (n,o) or a wing disc of 72h AEL cultured for 10 hours (p,q) stained as indicated. Scale bars are 10 μm. H/H fold (green arrow) and H/P fold (magenta arrow) are indicated.

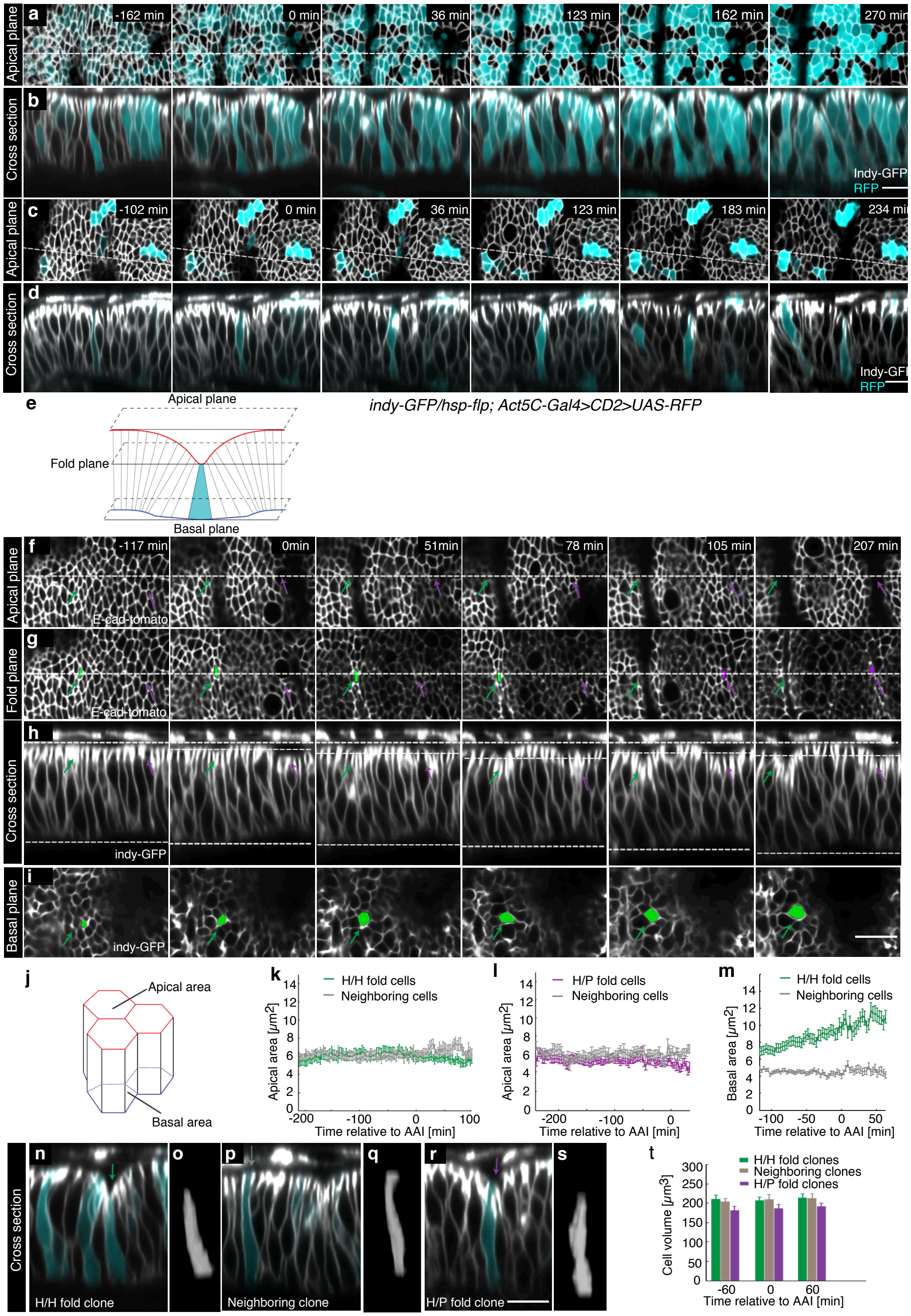

Supplementary Figure 2. Quantitative analysis of cell shape changes during fold formation

(a-d) Top view (a,c) and cross-sectional images (b,d) of a time-lapse movie of cultured wing discs expressing Indy-GFP (grey) in all cells and RFP (turquoise) in clones of cells of the H/H fold (a,b) or the H/P fold (c,d). Dotted lines in (a,c) indicate position of cross-section shown in (b,d). Scale bars are 10 µm.

(e) Scheme illustrating the apical-basal position of the apical, fold and basal planes shown in (f,g,i). Red line indicates position of adherens junctions. Blue line indicates basal surface of the tissue.

(f-i) Top views at the apical plane (f) or the fold plane (g), cross-sectional images (h) and top view on the basal plane (i) of a time-lapse movie of cultured wing discs expressing Indy-GFP and E-cadherin-tomato, a marker for adherens junctions. (f,g) show the E-cadherin-tomato channel; (h,i) show the Indy-GFP channel. In (g and i), exemplary H/H and H/P fold cells are highlighted in green and magenta, respectively. Green and magenta arrows indicate position of the H/H and H/P fold, respectively. Dotted lines in (f,g) indicate position of cross-section shown in (h). Dotted lines in (h) indicate position of apical plane, fold plane and basal plane shown in (f), (g) and (i), respectively. Scale bars are 10 µm.

(j) Scheme illustrating the apical and basal area of cells.

(k) Apical cross sectional area of H/H fold cells and neighboring cells as a function of time relative to first appearance of apical indentation (AAI) is shown. Mean and s.e.m. are shown. n= 12 cells of 3 wing discs for the H/H fold and n= 12 cells of 3 wing discs for neighboring cells.

(l) Apical cross sectional area of H/P fold cells and neighboring cells as a function of time relative to first appearance of apical indentation (AAI) is shown. Mean and s.e.m. are shown. n= 20 cells of 3 wing discs for the H/P fold and n= 12 cells of 3 wing discs for neighboring cells.

(m) Basal cross sectional area of H/H fold cells and neighboring cells as a function of time relative to first appearance of apical indentation (AAI) is shown. Mean and s.e.m. are shown. n= 30 cells of 3 wing discs for the H/H fold and n= 30 cells of 3 wing discs for neighboring cells.

(n-s) Cross-sectional images of time-lapse movies of cultured wing discs expressing Indy-GFP (grey) in all cells and RFP (turquoise) in clones of cells of the H/H fold (n,o), neighboring cells (p,q) or the H/P fold (r,s). (o,q,s) show views of a 3D-rendering of the clones. Scale bars are 10 μm.

(t) Cell volume of H/H fold cells, neighboring cells or H/P fold cells as a function of time relative to first appearance of apical indentation (AAI) is shown. Mean and s.e.m. are shown. n= 10 clones of 8 wing discs for the H/H fold, n= 10 clones of 4 wing discs for neighboring cells and n= 10 clones of 7 wing discs for the H/P fold. For comparisons between different time points, $p>0.05$, Student's t-test.

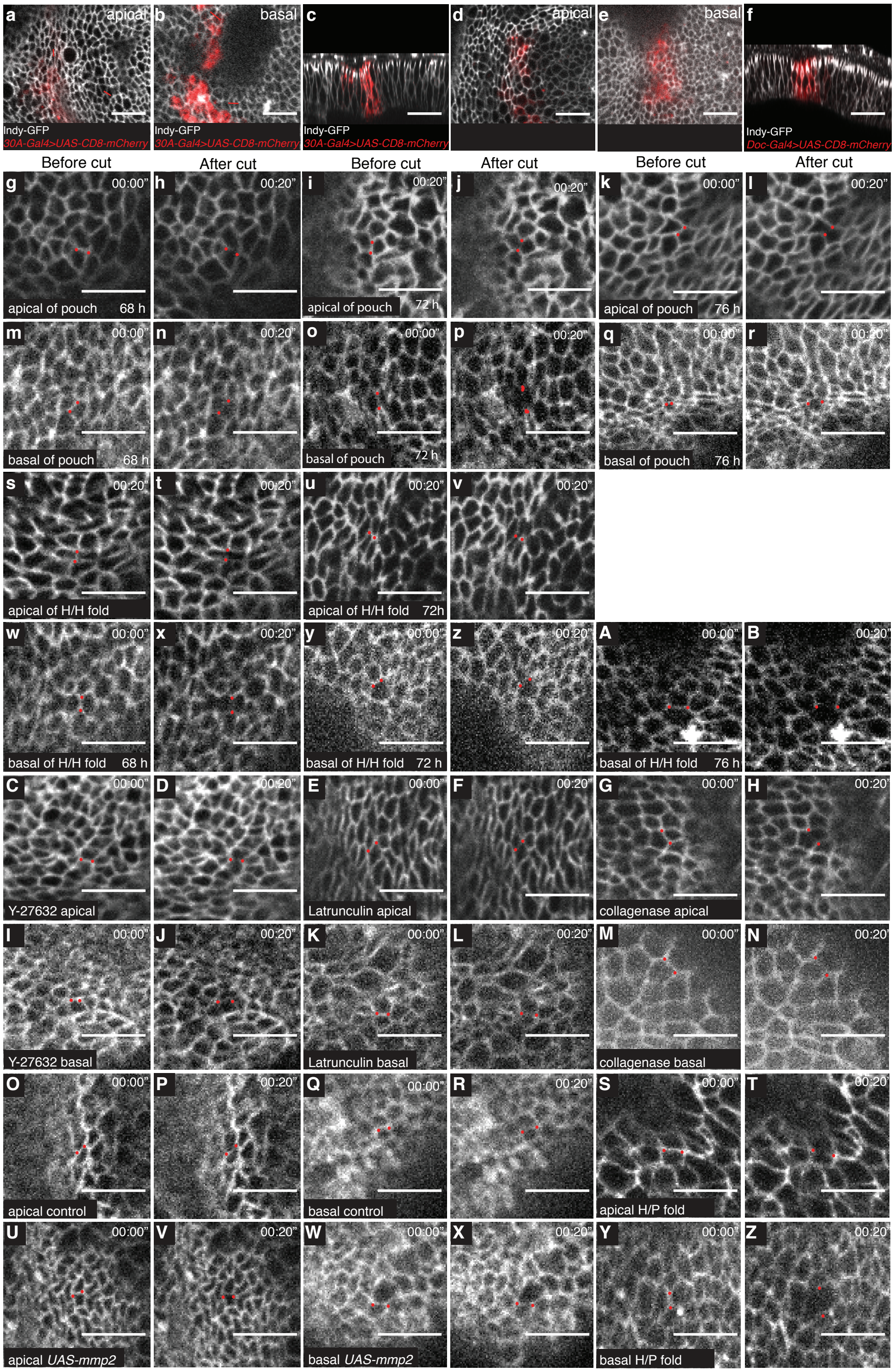

Supplementary Figure 3. Laser ablations

(a-c) Apical, basal and cross-sectional views of a wing disc of a 72h AEL larva expressing CD8-mCherry (red) under control of the 30A-Gal4 line (*30A-Gal4,*

UAS-CD8-mCherry). Indy-GFP marks cell outlines (grey). *30A*-Gal4 is active in cells of the H/H fold. Scale bars are 10 µm.

(d-f) Apical, basal and cross-sectional views of a wing disc of a 76h AEL larva expressing CD8-mCherry (red) under control of the doc-Gal4 line (*doc-Gal4, UAS-CD8-mCherry*). Indy-GFP marks cell outlines (grey). *doc*-Gal4 is active in cells of the H/P fold. Scale bars are 10 µm.

(g-Z) Wing disc cells of larvae of the indicated times after egg lay expressing Indy-GFP before and 20 seconds after ablation of a single cell edge at the apical or basal side of the epithelium. The region of the wing disc in which the ablated cell edge localized and genetic and drug perturbations are indicated. Scale bars are 10 µm.

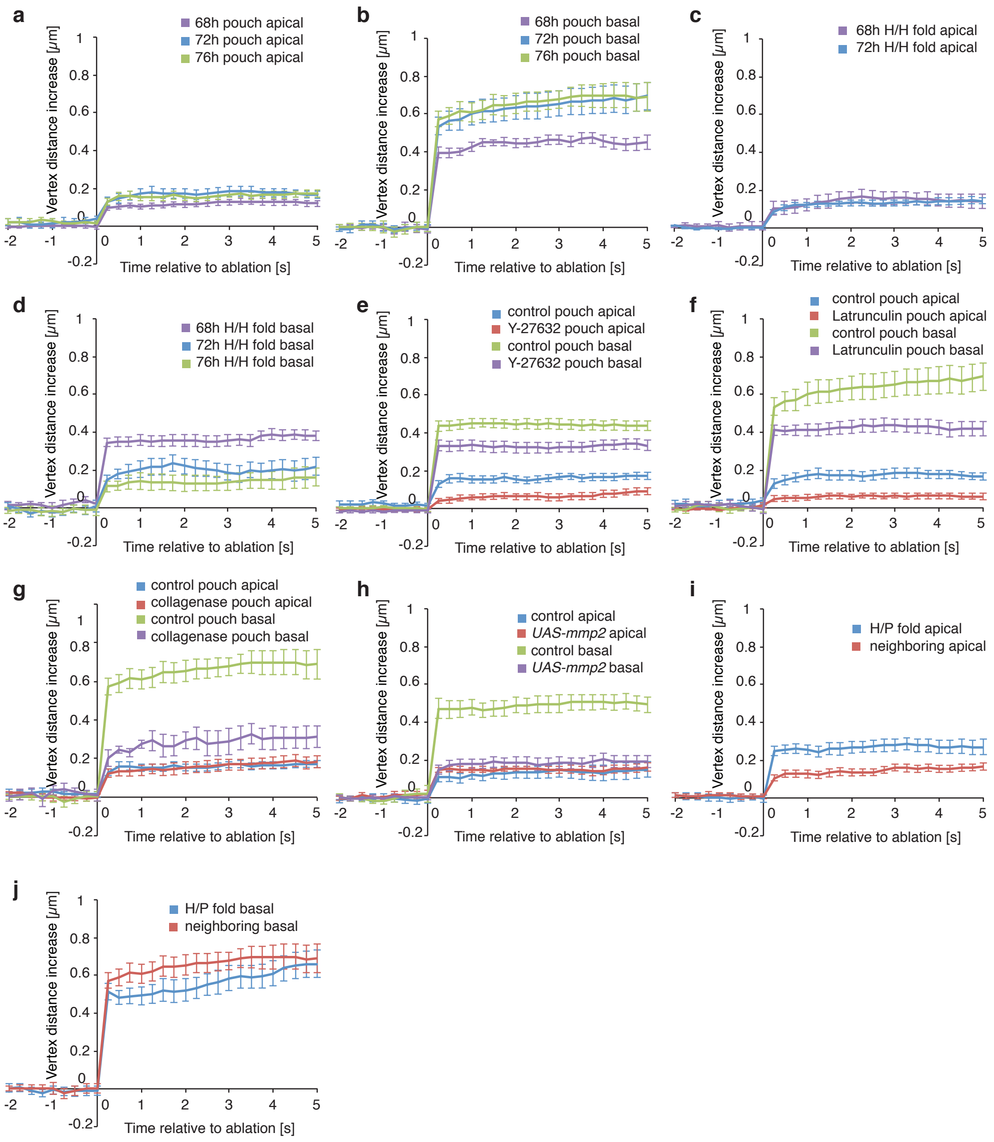

Supplementary Figure 4. Quantitative analysis of laser ablation experiments

(a-j) Change in distance between the two vertices of apical or basal cell edges upon ablation of the cell edge as a function of time relative to ablation for the indicated regions of the wing discs, age of larva (AEL) and genetic and drug perturbations. Mean and s.e.m. are shown (n= 15 cuts for each experiment).

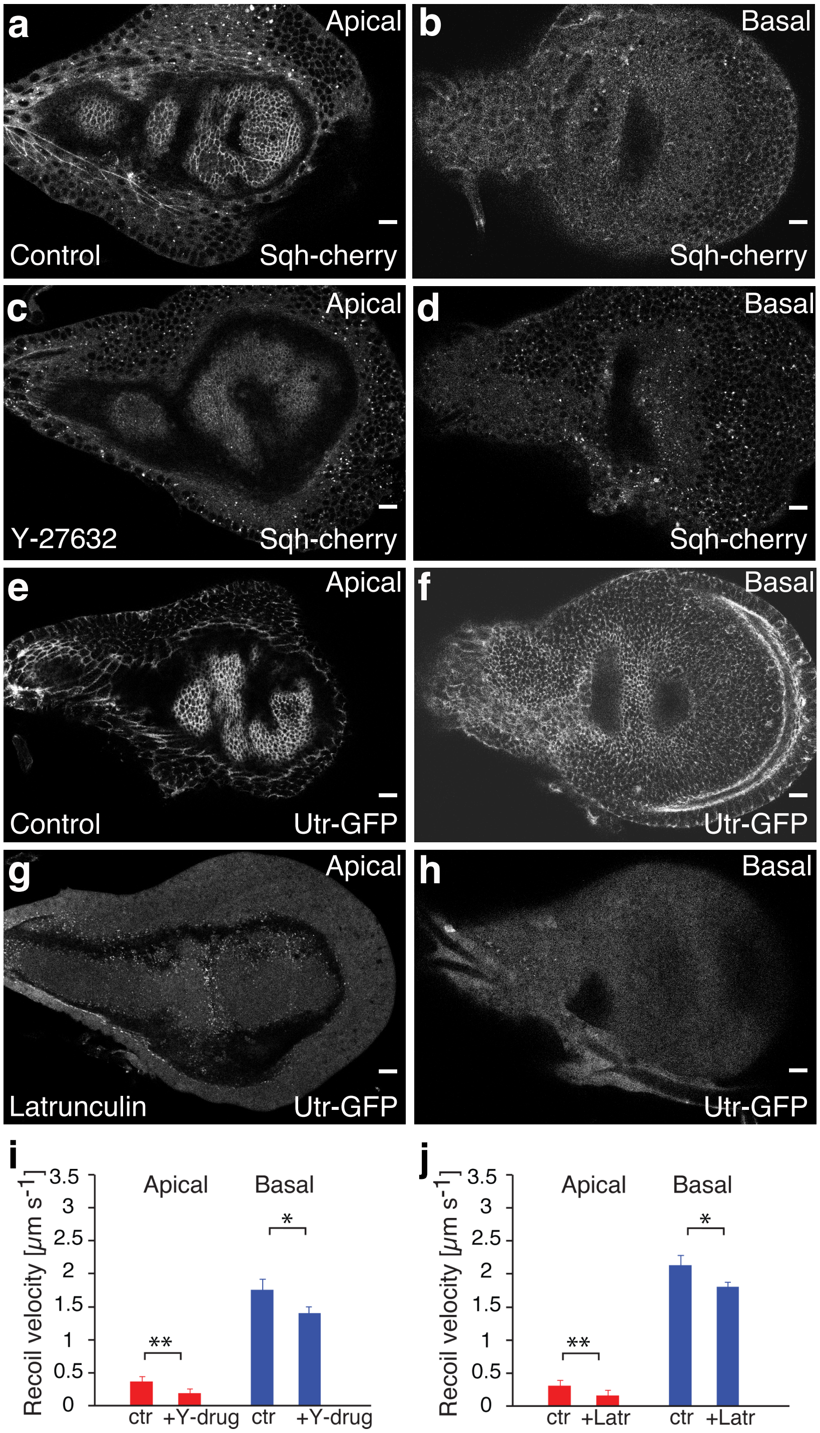

Supplementary Figure 5. Actomyosin contributes to basal edge tension

(a-d) Apical and basal views of 76h AEL wing discs expressing Sqh-cherry. Wing discs were incubated in culture medium (control, a,b) or culture medium containing 1mM Y-27632 for 60 min (c,d) prior to imaging. Scale bars are 10 μm.

(e-h) Apical and basal views of 76h AEL wing discs expressing Utr-GFP. Wing discs were incubated in culture medium (control, e,f) or culture medium containing 4 μM latrunculin for 60 min (g,h) prior to imaging. Scale bars are 10 μm.

(i) Average recoil velocity of the two vertices at the end of an ablated cell edge in the pouch region, within 250 ms after ablation, for 72h AEL wing discs. Recoil velocities are shown for ablations of apical and basal cell edges and for wing discs incubated in culture medium (control) or culture medium containing 1mM Y-27632 (Y-drug) for 60 min. prior to ablation, as indicated. Mean and s.e.m. are shown (n=15 cuts) (**: p<0.01; *: p<0.05, Student's *t*-test).

(j) Average recoil velocity of the two vertices at the end of an ablated cell edge in the pouch region, within 250ms after ablation, for 72h AEL wing discs. Recoil velocities are shown for ablations of apical and basal cell edges and for wing discs incubated in culture medium (control) or culture medium containing 4 μM Latrunculin (Latr.) for 60 min. prior to ablation, as indicated. Mean and s.e.m. are shown (n=15 cuts) (**: p<0.01; *: p<0.05, Student's *t*-test).

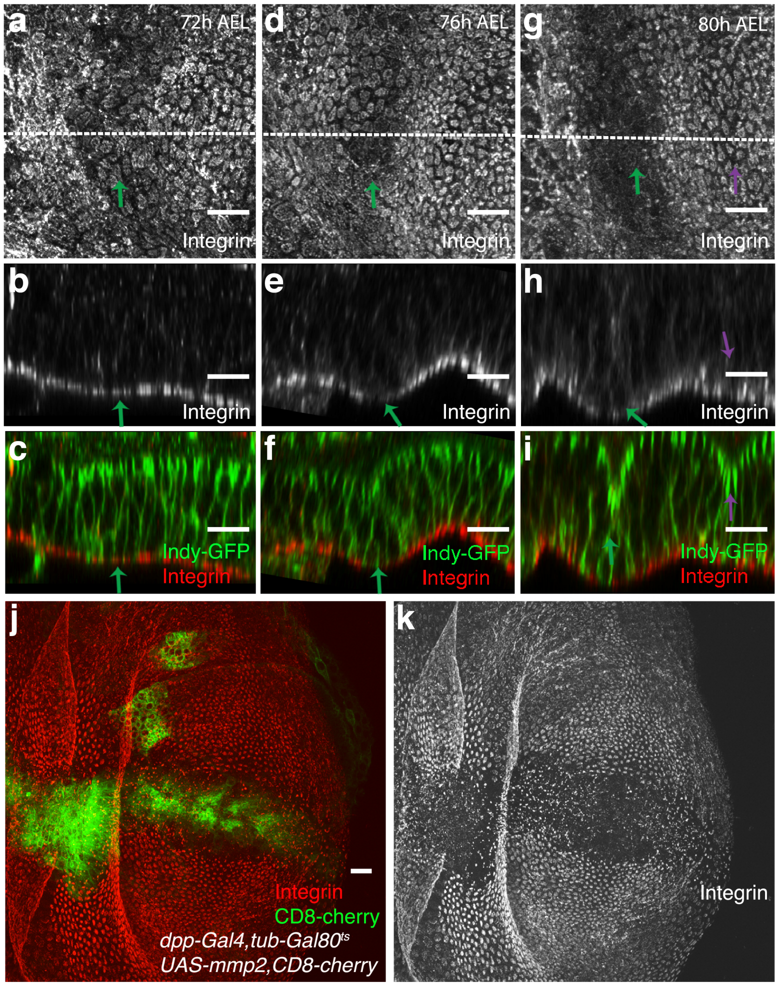

Supplementary Figure 6. Integrin levels are reduced in H/H, but not H/P fold cells, and in cells expressing MMP2

(a-i) Basal (a,d,g) and cross-sectional (b,c,e,f,h,i) views of Indy-GFP-expressing wing discs of larvae of the indicated time points stained for βPS-Integrin. Scale bars are 10μm. H/H fold (green arrow) and H/P fold (magenta arrow) are indicated.

(j-k) Basal view of a wing disc expressing MMP2 in a stripe of cells under control of *dpp-Gal4* labeled by expression of CD8-cherry (green). Integrin staining is shown in red (j) or grey (k). Larvae were incubated for 12 h at 29°C before dissection to induce MMP2 expression. Scale bar is 10 μm.

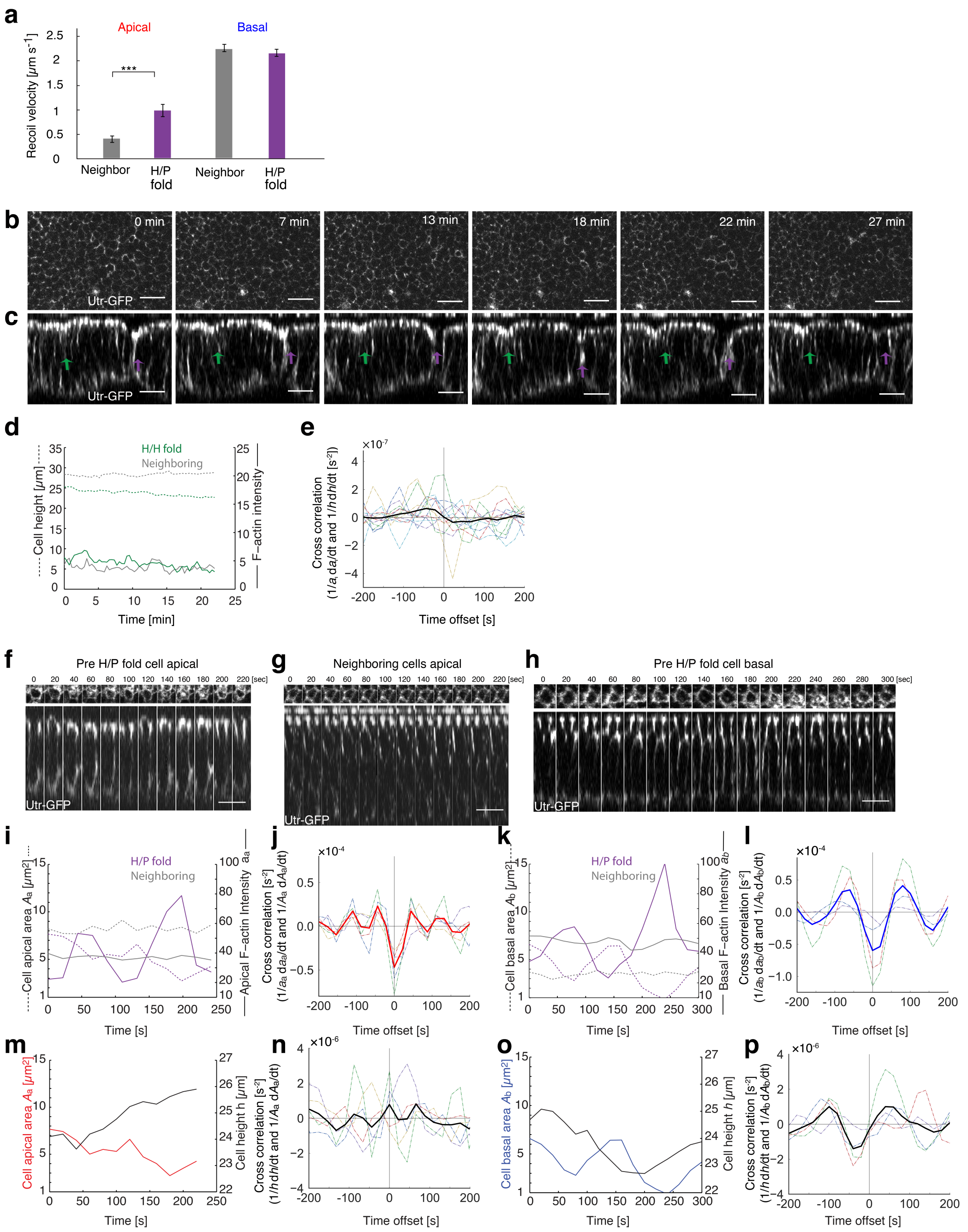

Supplementary Figure 7. Quantifications of fold cell shapes and F-actin intensity

(a) Average recoil velocity of the two vertices at the end of an ablated cell edge of H/P fold cells and neighboring cells, within 250ms after ablation. Recoil velocities are shown for ablations of apical and basal cell edges, as indicated. Mean and s.e.m. are shown (n=15 cuts)(***: $p < 0.001$, Student's *t*-test).

(b, c) Top view (b) and cross-sectional (c) images of a time-lapse movie of a 76h AEL cultured wing disc expressing Utr-GFP to visualize F-actin. Green and magenta arrows mark H/H and H/P fold, respectively. Scale bars are 10 μm.

(d) Cell height and lateral F-actin intensity measured simultaneously in a H/H pre-fold cell and in a neighbouring cell as a function of time with t=0 corresponding to the beginning of the measurement.

(e) Cross-correlation of the relative rate of change of lateral F-actin intensity $(1/a_{\mathrm{l}}) da_{\mathrm{l}}/dt$ and relative rate of height change $(1/h)\ dh/dt$ in H/H fold cells. N= 12 cells. Black line shows the average cross correlation.

(f-h) Apical views of pre H/P fold (f) or neighboring (g) cells or basal views of pre H/P fold cells (h) of time-lapse movies of 76 h AEL cultured wing discs expressing Utr-GFP. Lateral views are shown below. Scale bars are 10 μm.

(i) Apical cell area A_{a} and apical medial F-actin intensity a_{a} in the H/P fold (magenta) and neighboring cells (grey) as a function of time.

(j) Cross-correlation of relative rate of change of apical medial F-actin intensity $(1/a_{\mathrm{a}}) da_{\mathrm{a}}/dt$ and relative rate of apical area change $(1/A_{\mathrm{a}})\ dA_{\mathrm{a}}/dt$ in H/P fold cells. N= 5 cells. Red line shows the average cross correlation.

(k) Basal cell area A_{b} and basal medial F-actin intensity a_{b} in the H/P fold (magenta) and neighboring cells (grey) as a function of time.

(l) Cross-correlation of relative rate of change of basal medial F-actin intensity $(1/a_{\mathrm{b}}) da_{\mathrm{b}}/dt$ and relative rate of basal cell area change $(1/A_{\mathrm{b}}\ ) dA_{\mathrm{b}}/dt$ in H/P fold cells. N= 4 cells. Blue line shows the average cross correlation.

(m) Apical cell area A_{a} and cell height h of a H/P fold cell as a function of time.

(n) Cross-correlation of relative rate of height change $(1/h)\ dh/dt$ and relative rate of cell apical area change $(1/A_{\mathrm{a}})\ dA_{\mathrm{a}}/dt$ in H/P fold cells. N= 5 cells. Black line shows the average cross correlation.

(o) Basal cell area A_{b} and cell height h of a H/P fold cell as a function of time.

(p) Cross-correlation of relative rate of height change $(1/h)\ dh/dt$ and relative rate of basal area change $(1/A_{\mathrm{b}}\ )dA_{\mathrm{b}}/dt$ in H/P fold cells. N= 4 cells. Black line shows the average cross correlation. Cross-correlation coefficient at peak is -0.29, compared to the cross-correlation coefficient at peak of -0.65 in Fig. S7l.

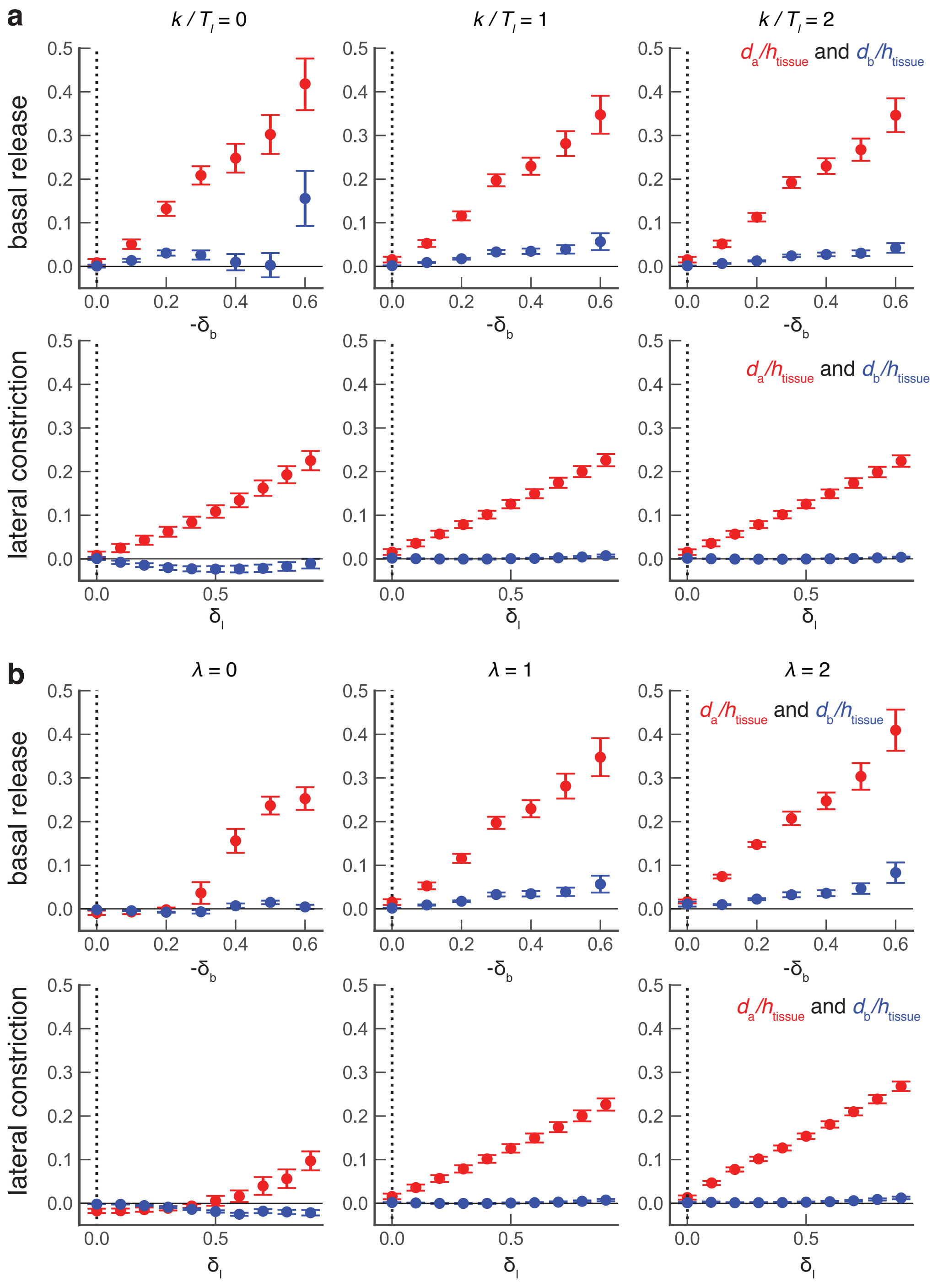

Supplementary Figure 8. Effect of variation in mechanical parameters on simulations of fold formation

Relative apical and basal indentations (d_a/h_{tissue}, d_b/h_{tissue}) driven by a decrease in basal surface tension (upper rows in (a) and (b)) and an increase in

lateral surface tension (lower rows) in a stripe of cells. Average deformations are shown with standard error of the mean obtained from N=4 simulations. The mechanical parameters were chosen to be the same as in Figure 7c (see Supplementary Methods for additional details), except for two mechanical parameters which were varied independently: in the simulations in (a) the attachment stiffness k/T_l was varied around $k/T_l = 1$, while in the simulations in (b) the ratio λ between line and surface tensions was varied around $\lambda=1$. While the strength of the deformations varies quantitatively for the different parameter sets, we find that the qualitative behavior of the deformation as a function of surface and line tension changes is preserved when varying these two parameters.

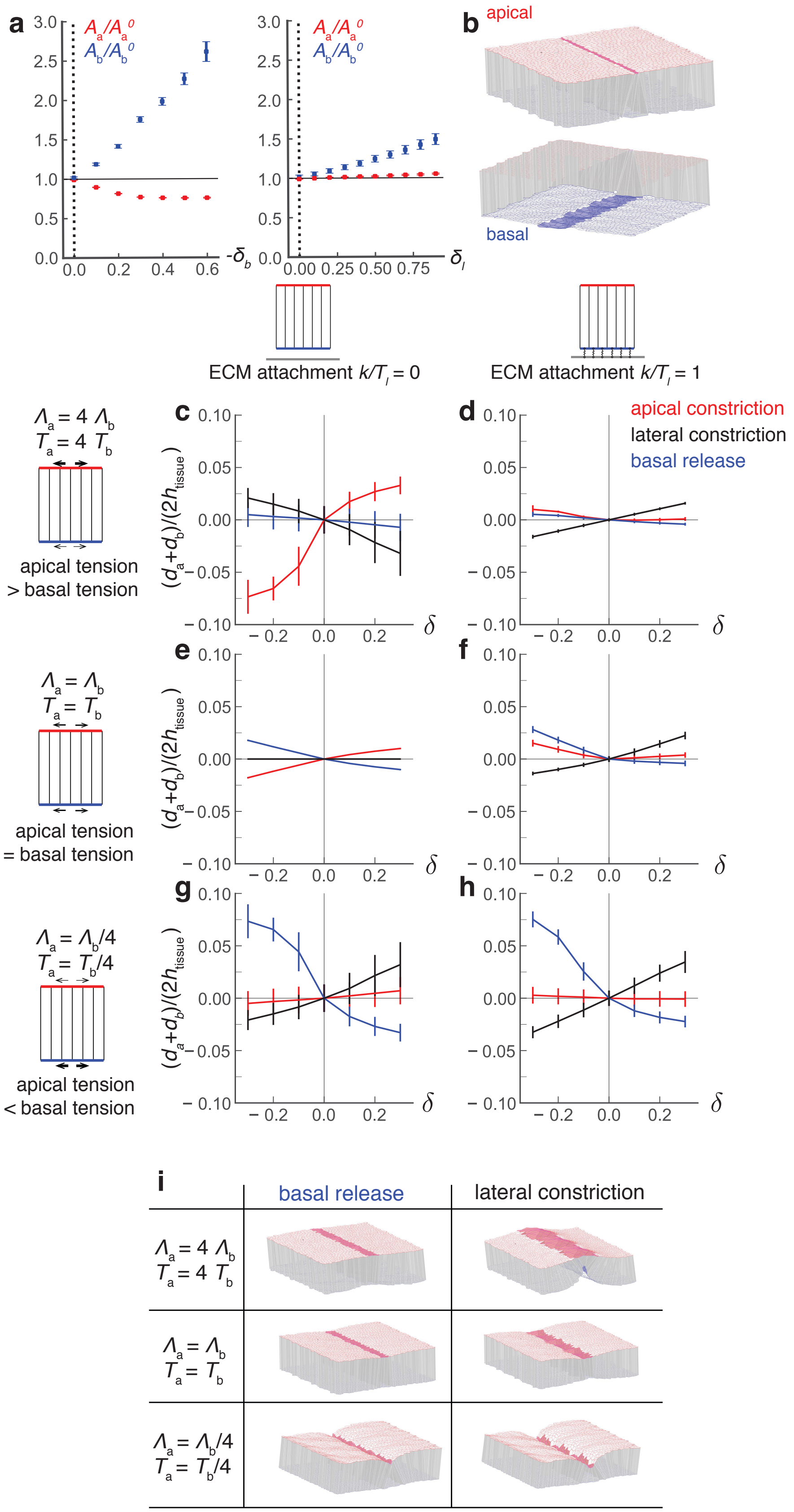
a
b
apical
basal
ECM attachment $k/T_l = 0$
ECM attachment $k/T_l = 1$
c
d
e
f
g
h
apical constriction
lateral constriction
basal release
$\Lambda_a = 4\ \Lambda_b$
$T_a = 4\ T_b$
apical tension
> basal tension
$\Lambda_a = \Lambda_b$
$T_a = T_b$
apical tension
= basal tension
$\Lambda_a = \Lambda_b/4$
$T_a = T_b/4$
apical tension
< basal tension
$(d_a+d_b)/(2h_{tissue})$
i
basal release
lateral constriction

Supplementary Figure 9. Effect of asymmetry in apical and basal surface tension and ECM attachment on simulations of fold formation

(a) Average apical and basal surface areas (A_{a}, A_{b}) of cells within the fold normalized with the average apical and basal cell areas of all cells before folding (A_{a}^{0}, A_{b}^{0}) as a function of the mechanical changes in the fold cells, for the simulations shown in Figure 7c-d. The average apical area decreases slightly following the basal tension decrease and is nearly constant following the lateral tension increase (compare with Supplementary Figure 2k,l).

(b) Apical and basal view of 3D vertex model simulations showing deformations resulting from increased apical tension in a stripe of cells. Initial conditions prior to fold formation are obtained with $T_{\mathrm{a}} = T_{\mathrm{b}}, \Lambda_{\mathrm{a}} = \Lambda_{\mathrm{b}}$. Apical edge and surface tensions T_{a} and Λ_{a} are then increased by a factor 5 in a 4-cell wide stripe, and the images show the resulting equilibrium shape. While the apical surface areas of the constricting cells are strongly reduced, the strength of the apical indentation remains small.

(c-h) Effect of initial asymmetry of apical and basal tensions on simulated folds. Simulations are performed by introducing a stripe of cells with modified mechanical parameters, as described in the Supplementary Methods, in a tissue with similar contributions from surface and line tensions ($\lambda = 1$). Three qualitatively different perturbations within the stripe are considered: increase in apical surface and line tensions (red), decrease in basal surface and line tensions (blue), or increase in lateral surface tension (black). The normalized displacement of the epithelial midplane $(d_{\mathrm{a}} + d_{\mathrm{b}})/(2h_{\mathrm{tissue}})$ with d_{a} the apical indentation, d_{b} the basal deformation and h_{tissue} the average cell height before folding, is plotted with the s.e.m. (N=4) as a function of the relative increase in

apical tension and lateral tension and relative decrease in basal tension (red curves: $\delta = \delta_{\mathrm{a}}$, black curves: $\delta = \delta_{\mathrm{l}}$, blue curves: $\delta = \delta_{\mathrm{b}}$). In (d, f, h) basal vertices are attached to an external substrate mimicking attachment to the ECM, and this constraint is released in (c, e, g). In (c, d), apical surface and line tensions are initially 4 times larger than basal surface and line tensions. In (e, f), basal and apical surface and line tensions are initially equal. In (e), the mechanical tissue properties are initially symmetric with respect to exchange of apical and basal sides. In that case, an increase in lateral surface tension does not result in net folding. In (g, h), basal surface and line tensions are initially 4 times larger than apical surface and line tensions. Both with and without ECM attachment, the initially larger basal surface and line tensions favor a folding of higher magnitude for basal tension decrease relative to increased apical tension. (i) Examples of 3D vertex model simulations with varying asymmetry in apical and basal tensions, prior to folding. The left column shows the resulting deformation for a 40% decrease in basal surface and line tensions, while the right column shows the effect of a 2-fold increase of lateral surface tension in a 4-cell wide stripe in the epithelium. The ECM attachment is set to zero throughout the tissue ($k = 0$), the apical and basal line tensions are comparable to the surface tensions ($\lambda = 1$), and the relative strength of apical to basal tensions is varied in the 3 rows.

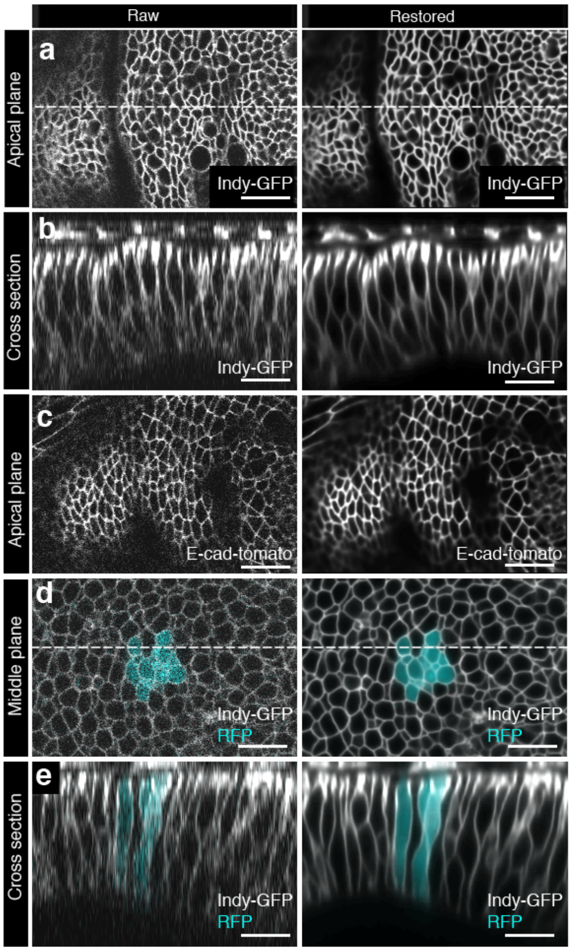

Supplementary Figure 10. Denoising and restoration of axial resolution of acquired volumes.

(a,b) Top view (a) and cross-sectional (b) images of a time-lapse movie of a cultured wing disc expressing Indy-GFP. Scale bars are 10 μm.

(c) Top view images of a time-lapse movie of a cultured wing disc expressing E-cad-tomato. Scale bars are 10 μm.

(d,e) Middle plane (d) and cross-sectional (e) images of a time-lapse movie of a cultured wing disc expressing Indy-GFP (grey) in all cells and RFP (turquoise) in clones of cells. Scale bars are 10 μm.

Raw images are shown to the left; images after restoration applying CARE are shown to the right.

Supplementary Methods

3D vertex model for epithelial mechanics

In this section we briefly describe the 3D vertex model for epithelial mechanics [1] used for simulations of epithelial folding.

Geometry of the 3D vertex model

In the 3D vertex model the tissue is represented by a set of apical and basal vertices, associated with the apical and basal surfaces of the epithelium. Vertices correspond to the intersection of three or more cells on the tissue apical or basal surfaces. Here for simplicity we have enforced apical and basal tissue topologies to be identical. The vertices forming an apical, basal or lateral surface are not necessarily coplanar. In order to define the surface enclosing a cell, each cell boundary is defined by triangulation of the vertices on the contour of the boundary. The triangulation is obtained by joining neighbouring vertices on the contour with the center of mass of the surface contour. Each cell is therefore enclosed by a set of triangles (Figure 7a) which allow to define the cell volume, and apical, basal and lateral surface areas.

Vertices move in space according to forces acting on them, as described in the sections below. In addition, the tissue topology is allowed to change through topological transitions involving neighbour exchange [1].

In simulations discussed here, the tissue is assumed to be confined in a periodic box in x and y direction, with system size L_x and L_y respectively. The lengths L_x and L_y are taken as degrees of freedom of the system.

Virtual mechanical work

The internal differential virtual work for the 3D vertex model is written

$$\delta W_i = \sum_k T_k \delta A_k + \sum_{\langle i,j \rangle} \Lambda_{ij} \delta l_{ij} - \sum_\alpha P_\alpha \delta V_\alpha, \tag{1}$$

where the index k label interfaces, the indices i, j label vertices, and the index α labels cells. The second sum runs over junctions connecting vertices $\langle i, j \rangle$. The volume of cell α, the surface area of interface k and the length of the junction joining the vertices i and j are denoted V_α, A_k, and l_{ij} respectively. The pressure acting in cell α is denoted P_α, the surface tension on interface k, T_k, and the line tension acting on the junction $\langle i, j \rangle$, Λ_{ij}.

In general tensions generated on cellular interfaces and along junctions between cells can depend on the surface area of the interface or the length of the junction (i.e. $T_k = T_k(A_k)$, $\Lambda_{ij} = \Lambda_{ij}(l_{ij})$). For simulations of fold formation, we assume that apical and basal surfaces have a constant tension if their area is larger than a preset threshold area, and have a linearly elastic behaviour below this threshold, with surface elasticity constant $K_{2\text{D}}$:

$$T_k(A_k) = \begin{cases} T_k^0 & \text{for } A_k > A^0 \\ T_k^0 + K_{2\text{D}}(A_k - A^0) & \text{for } 0 \leq A_k < A^0. \end{cases} \tag{2}$$

We choose here $A^0 = \bar{A}$, with $\bar{A}$ the average apical surface area of wild-type cells prior to fold formation. We have introduced an elastic contribution with elastic modulus $K_{2\text{D}}$ to prevent collapse of apical or basal surface areas. The surface tensions acting along lateral surfaces are taken to be constant, independent of the surface area. Apical and basal line tensions on the cell bonds are constant for bond lengths larger than 1% of their equilibrium length, defined by the bond length of the equilibrated hexagonally

packed tissue with the same mechanical parameters, and for smaller bond lengths they go linearly to zero:

$$\Lambda_{ij}(l_{ij}) = \begin{cases} \Lambda_{ij} & \text{for } l_{ij} > l_{ij}^0/100 \\ \frac{l_{ij}}{l_{ij}^0/100}\Lambda_{ij} & \text{for } l_{ij} \le l_{ij}^0/100. \end{cases} \tag{3}$$

This length dependent tension was introduced to prevent rare cases of numerical oscillations of bond lengths for short bonds, for the case where T1 transitions cannot occur because the corresponding bond on the opposite apical/basal side is not sufficiently shrunk.

The cell pressure P_α is taken to be linear in the deviation of the volume from the preferred volume,

$$P_\alpha = -K_{3\text{D}}(V_\alpha - V^0), \tag{4}$$

where V^0 is the preferred cell volume and the proportionality constant $K_{3\text{D}}$ is a bulk elastic modulus.

In addition, we introduce external basal springs that represent tissue attachment to the extracellular matrix (ECM). The ECM is considered to be a flat surface located at $z = 0$. Attachment to the ECM is taken into account through an external differential virtual work which reads

$$\delta W_e = \sum_v k x_v \delta x_v, \tag{5}$$

where the shortest distance of a vertex v to the ECM is denoted x_v, and the sum is taken over basal vertices v. The spring constant k represents elastic bonds attaching the tissue to the extracellular matrix. With the choice of virtual work made in Sup-

plementary Equation 5, we consider elastic bonds that prevent normal deformations away from the ECM but are free to slide tangentially to the plane of the ECM. The total differential virtual work function then reads:

$$\delta W = \delta W_i + \delta W_e. \tag{6}$$

Resulting forces

The force $\mathbf{F}_i$ that acts on vertex i with position $\mathbf{x}_i$ can be obtained by taking the derivative of the virtual work with respect to the vertex position:

$$\mathbf{F}_i = -\frac{\delta W}{\delta \mathbf{x}_i}. \tag{7}$$

Note that when calculating the change in virtual work resulting from the variation of a vertex position in Supplementary Equation 7, we include the variations in the positions of the centres of mass of surfaces that arise when a vertex of the contour of the surface is displaced [1]. The tissue is in mechanical equilibrium if the forces on all vertices i vanish:

$$\mathbf{F}_i = \mathbf{0}. \tag{8}$$

Relaxation to mechanically equilibrated shapes

For the choice of the virtual work function we use here, δW can be integrated to yield

$$W = \sum_\alpha -P_\alpha V_\alpha + \sum_k W_k(A_k) + \sum_{ij} W_{ij}(l_{ij}) + \sum_v \frac{k}{2} x_v^2, \tag{9}$$

where $W_k(A_k) = \int_0^{A_k} T_k(\tilde{A}_k) d\tilde{A}_k$ and $W_{ij}(l_{ij}) = \int_0^{l_{ij}} \Lambda_{ij}(\tilde{l}_{ij}) d\tilde{l}_{ij}$. For a fixed topology of the network of junctions and vertices, the work W can be minimised with respect to the position of the vertices and the system size L_x, L_y to find mechanically equilibrated

configurations of the network. This is done numerically in a C++ implementation of the Polak-Ribiére conjugate gradient algorithm [2].

Simulations of epithelial folding in the 3D vertex model

Starting configurations

Simulations are performed with an epithelium containing 500 cells in a periodic box, containing about 33 cells along the x-direction and 15 cells along the y-direction (Figure 7b). To obtain different starting configurations of the tissue, random tissues are created by performing a 2D Voronoi tessellation of randomly distributed points in a periodic box of size $L_x^0 \times L_y^0$ with $L_x^0/L_y^0 = 2$. Apical and basal vertices are then assigned the same x and y coordinates, but different z coordinates. This random initial configuration is relaxed to a mechanically equilibrated shape by minimising the work function with respect to system size and vertex positions, and allowing for neighbour exchange events. For each set of examined mechanical parameters, we ran simulations with 4 or more different starting configurations of the tissue.

Mechanical parameters prior to fold formation

Description	Parameter
cell volume	$V_0/l_0^3 = 1$
lateral surface tension	$T_{\rm l}/T_{\rm l} = 1$
volume elasticity	$K_{\rm 3D}/(T_{\rm l}/l_0^4)$
apical surface tension	$T_{\rm a}/T_{\rm l}$
basal surface tension	$T_{\rm b}/T_{\rm l}$
apical line tension	$\Lambda_{\rm a}/(l_0 T_{\rm l})$
basal line tension	$\Lambda_{\rm b}/(l_0 T_{\rm l})$
stiffness of ECM attachment	$k/T_{\rm l}$
apical and basal area elasticity	$K_{\rm 2D}/(T_{\rm l}/l_0^2)$

Table 1: List of normalized mechanical parameters in 3D vertex model simulations, determining the tissue state prior to fold formation.

We discuss here mechanical parameters used to describe the tissue prior to fold

formation. Mechanical parameters are set to be equal in all cells. Cells have a preferred volume V_0, apical, basal and lateral surface tensions T_a, T_b, T_l, apical and basal line tensions Λ_a, Λ_b, volume elasticity $K_{3\text{D}}$, surface elasticity $K_{2\text{D}}$, and a stiffness of basal vertices attachment to the ECM k. The corresponding parameters are listed in TABLE 1, where we use the length scale $l_0 = V_0^{\frac{1}{3}}$ and the lateral surface tension T_l for normalisation. The volume elasticity is chosen such that $K_{3\text{D}} l_0^4/T_\text{l} \gg 1$, such that the volume of the cells remains practically constant. 6 free mechanical adimensional parameters then remain: T_a/T_l, T_b/T_l, $\Lambda_\text{a}/(l_0 T_\text{l})$, $\Lambda_\text{b}/(l_0 T_\text{l})$, k/T_l and $K_{2\text{D}}/(T_\text{l}/l_0^2)$. In order to restrain our parameter search, we assume that the normalised ratio between surface and line tensions, λ, is the same apically and basally:

$$\lambda = \frac{\Lambda_\text{a}}{T_\text{a} l_0} = \frac{\Lambda_\text{b}}{T_\text{b} l_0}. \tag{10}$$

In addition, we further constrain parameters by requesting that the aspect ratio of simulated cells matches the wing disc cells aspect ratio before fold formation. In simulations we define the average aspect ratio of a flat packing by $\beta_\text{sim} = h/\sqrt{A}$, with h the cell height and A the average apical or basal area. The aspect ratio of simulated cells β_sim can be estimated by solving the following equation, obtained by calculating the preferred shape of a flat, regular packing of hexagonal cells with mechanical parameters as specified above [1]:

$$\beta_\text{sim} \simeq \frac{\sqrt{2}}{3^{\frac{1}{4}} T_\text{l}} \left((T_\text{a} + T_\text{b}) + \frac{3^{1/4}}{\sqrt{2}} \beta_\text{sim}^{1/3} \frac{\Lambda_\text{a} + \Lambda_\text{b}}{l_0} \right). \tag{11}$$

To estimate the aspect ratio of the cells in the imaginal wing disc, we evaluated the average ratio of cell height h to cell cross-sectional length $(l_a + l_b)/2$, at 68 hAPF, in cross-sections perpendicular to the folds and outside the fold ($N = 4$ wing discs). We found an average ratio $h/((l_a + l_b)/2) \simeq 16$. Note that epithelial cells are anisotropic in the plane of epithelium, but for simplicity we ran simulations with an isotropic cellular

packing. We then require that the simulated aspect ratio in the equation above is equal to this experimentally measured ratio:

$$\beta_{\text{sim}} = 16. \quad (12)$$

Taking into account this constraint, we find non-zero values of lateral surface tension T_{l}. A very low recoil velocity is observed following ablation of lateral interfaces of cells neighbouring the H/P fold (Figure 6j). It seems however unlikely to us that no tension is exerted along the lateral interfaces, as actin and myosin can be observed on these interfaces (Figure 3a-h), and the shape of interfaces appear visually consistent with interfaces under tensions perturbed by nuclei (see e.g. Figure 5c).

In addition, laser cutting experiments described in Figure 3i-m show that the average recoil velocity of laser ablated basal junctions is on average about four times higher than the average recoil velocity of apical junctions. Assuming that the frictions and viscosities of the actin apical and basal cortices are similar, these measurements suggest that the basal tension is four times higher than the apical tension. This observation is taken into account in the 3D vertex model simulations by requiring:

$$\alpha = \frac{T_{\text{b}}}{T_{\text{a}}} = \frac{\Lambda_{\text{b}}}{\Lambda_{\text{a}}} = 4. \quad (13)$$

Simulation of epithelial folding

To simulate folding, a pre-fold region is manually defined by introducing a stripe of width $N \simeq 4$ in the relaxed homogeneous tissue. The mechanical parameters of cells within this stripe are subsequently changed according to the mechanisms of apical tension increase, basal tension decrease and lateral tension increase.

To simulate the mechanism of apical tension increase, the apical surface tension of cells in the stripe is changed to $T_{\text{a}}(1+\delta_{\text{a}})$ and the apical line tension is changed to $\Lambda_{\text{a}}(1+\delta_{\text{a}})$.

The basal tension decrease mechanism is simulated by introducing a change of basal surface tensions of cells in the stripe $T_\mathrm{b}(1+\delta_\mathrm{b})$, and in basal line tensions $\Lambda_\mathrm{b}(1+\delta_\mathrm{b})$. For both apical tension increase and basal tension decrease, the line tensions between folding and non-folding cells are given by the arithmetic mean of the line tensions of folding cells and non-folding cells. To simulate the mechanism of lateral tension increase, the lateral surface tension between folding cells is changed to $T_\mathrm{l}(1+\delta_\mathrm{l})$. The lateral surface tension between folding and outside cells is given by the arithmetic mean of the lateral surface tensions of folding cells and non-folding cells. Following these changes, the tissue shape is relaxed quasistatically to mechanical equilibrium. In Figure 7c, we plot the tissue deformation as a function of the magnitudes of relative tension changes. The resulting deformations look qualitatively similar to experimental deformations as a function of time (Figure 1n-q). To further study the dynamics of fold formation however would require the introduction of dissipative contributions coming from the tissue or its surrounding environment. It would be interesting to explore whether cellular surface tensions and line tensions are suddenly changing in the fold, with the dynamics of folding controlled by dissipative processes, whether active surface and line tensions are progressively changing as the fold indents with little viscous resistance to deformation, or whether an intermediate situation applies where both the dynamics of changes in active surface and line tensions and viscous resistance to motion controls the dynamics of fold formation.

Quantification of tissue deformation

To compare the mechanical equilibrium shapes in simulations to the shapes obtained in experiments, we calculate the shape parameters $d_\mathrm{a}/h_\mathrm{tissue}$, $d_\mathrm{b}/h_\mathrm{tissue}$, $l_\mathrm{a}/h_\mathrm{tissue}$ and $l_\mathrm{b}/h_\mathrm{tissue}$ for simulated tissues, defined in a similar way as shape parameters determined from experimental images of folds (Figure 1i). Specifically, the average tissue height h_tissue is taken to be the average distance of apical and basal cell contour centers

$\mathbf{b}_\mathrm{a}^\alpha$ and $\mathbf{b}_\mathrm{b}^\alpha$, for non-folding cells:

$$h_\mathrm{tissue} = \frac{1}{N}\sum_\alpha |\mathbf{b}_\mathrm{a}^\alpha - \mathbf{b}_\mathrm{b}^\alpha|, \tag{14}$$

where the sum is taken over the N cells α outside the folding region.

The position of the apical and basal plane is obtained from the average z-positions of all apical and basal cell contour centers of non-folding cells, $\bar{z}_\mathrm{a}^\mathrm{NF}$ and $\bar{z}_\mathrm{b}^\mathrm{NF}$ respectively:

$$\bar{z}_\mathrm{a}^\mathrm{NF} = \frac{1}{N}\sum_\alpha z_\mathrm{a}^\alpha, \quad \bar{z}_\mathrm{b}^\mathrm{NF} = \frac{1}{N}\sum_\alpha z_\mathrm{b}^\alpha, \tag{15}$$

where the sum is taken over the N cells α outside the folding region, and z_a^α and z_b^α denote the z-coordinates of the apical and basal cell contours of cell α.

The apical and basal indentations are then defined as the signed maximal distance of apical (respectively basal) cell contour centers of cells in the fold from the apical (respectively basal tissue plane):

$$d_\mathrm{a} = \bar{z}_\mathrm{a}^\mathrm{NF} - z_\mathrm{a}^{\alpha_\mathrm{a}^\mathrm{max}}, \quad d_\mathrm{b} = \bar{z}_\mathrm{b}^\mathrm{NF} - z_\mathrm{b}^{\alpha_\mathrm{b}^\mathrm{max}}, \tag{16}$$

where $\alpha_\mathrm{a}^\mathrm{max}$ and $\alpha_\mathrm{b}^\mathrm{max}$ are the cells whose apical or basal cell contour centers are furthest from the apical and basal tissue planes, respectively:

$$\alpha_\mathrm{a}^\mathrm{max} = \arg\max_\alpha |\bar{z}_a^\mathrm{NF} - z_a^\alpha| \tag{17}$$

$$\alpha_\mathrm{b}^\mathrm{max} = \arg\max_\alpha |\bar{z}_b^\mathrm{NF} - z_b^\alpha|. \tag{18}$$

The average apical and basal cross-section lengths of cells in the fold, l_a and l_b respectively, are defined by:

$$l_\mathrm{a} = \frac{\bar{A}_\mathrm{a}^\mathrm{F}}{L_y/N_y}, \quad l_\mathrm{b} = \frac{\bar{A}_\mathrm{b}^\mathrm{F}}{L_y/N_y}, \tag{19}$$

where L_y denotes the extension of the periodic box in y-direction, N_y denotes the average number of cells along the y-direction and $\bar{A}^F_{\text{a}}$ and $\bar{A}^F_{\text{b}}$ denote the average apical and basal cell areas of the folding cells, respectively.

Parameter space exploration

To perform a parameter search, we note that imposing the conditions stated in Supplementary Equations 10, 12 and 13 leaves us with 3 free remaining model parameters characterizing the tissue before fold formation. We choose here as free parameters the ratio between line tension and surface tension λ, the normalised stiffness of attachment to the ECM k/T_{l}, and the normalized cell surface area elasticity $K_{\text{2D}} l_0^2/T_{\text{l}}$. We fixed the cell surface area elasticity to $K_{\text{2D}} l_0^2/T_{\text{l}} = 10$, which we chose to be large enough to prevent the collapse of any apical and basal surfaces prior to fold formation. We then simulated the effect of a change of basal surface tensions and lateral surface tension in a stripe of cells on the shape of the tissue, varying the parameters k/T_{l} and λ.

Basal laser cutting experiments suggest that the basal tensions are reduced by 60% or more in the H/H fold (Figure 5a, average of relative difference between H/H fold and pouch at 68h, 72h and 76h). Similarly, experimental results presented in Figure 6 indicate that lateral surface tensions are significantly increased within H/P pre-fold cells. Consequently simulations results are shown in Figure 7c with either decreased basal line and surface tensions up to 60%, or increased lateral surface tension by up to a factor ~ 1. The resulting mechanical equilibrium shapes are quantified as described in section "quantification of tissue deformation". In Figure 7c we show shape quantifications for:

$$\lambda = 1, \quad k/T_{\text{l}} = 1, \tag{20}$$

showing a good agreement with experimental shapes (1n-q). The other parameters then read as follows:

$$T_\mathrm{a}/T_\mathrm{l} \simeq 0.9 \tag{21}$$

$$T_\mathrm{b}/T_\mathrm{l} \simeq 3.6 \tag{22}$$

$$\Lambda_\mathrm{a}/(T_\mathrm{l} l_0) \simeq 0.9 \tag{23}$$

$$\Lambda_\mathrm{b}/(T_\mathrm{l} l_0) \simeq 3.6 \tag{24}$$

$$k/T_\mathrm{l} = 1 \tag{25}$$

$$K_\mathrm{2D}/(T_\mathrm{l}/l_0^2) = 10. \tag{26}$$

In this parameter regime the apical and basal line tensions have a similar effective contribution to cell mechanics as apical and surface tensions, and the ECM attachment stiffness is small compared to the total tensions generated apically and basally. Figure 7c and 7d show results of the simulations for the parameters given above, for varying strengths of basal tension reduction and lateral tension increase.

To test how our simulation results depend on the choice of the two free parameters λ and k/T_l, we have analyzed deformations obtained when values of these parameters are varied around $k/T_\mathrm{l} = 1$ and $\lambda = 1$. Corresponding results are shown in Supplementary Figure 8a and Supplementary Figure 8b, where the two parameters are varied independently from 0 to 2. The qualitative behaviour of the fold deformation as a function of changes in surface and line tensions is preserved. We found however that quantitative differences can be seen in the resulting deformations, in particular for the case without basal attachment ($k/T_\mathrm{l} = 0$) or with vanishing line tensions compared to the surface tensions ($\lambda = 0$). These differences do not affect our main conclusions.

Variation of the relative apical and basal tension

To further explore the effect of the relative values of apical and basal tension on the formation of simulated folds we ran additional simulations where prior to fold formation, apical surface and junctional tensions are equal to basal surface and junctional tensions ($T_\mathrm{a}/T_\mathrm{b} = \Lambda_\mathrm{a}/\Lambda_\mathrm{b} = 1$), and simulations where apical surface and junctional tensions are four times higher than basal surface and junctional tensions ($T_\mathrm{a}/T_\mathrm{b} = \Lambda_\mathrm{a}/\Lambda_\mathrm{b} = 4$).

We then performed simulation of fold formation as described previously. The deformations arising from apical tension increase, lateral tension increase and basal tension decrease are shown in Supplementary Figure 9, both with and without attachment to the underlying ECM ($k/T_\mathrm{l} = 0$ or $k/T_\mathrm{l} = 1$). We find that larger initial basal surface and line tensions ($T_\mathrm{b}/T_\mathrm{a} = \Lambda_\mathrm{b}/\Lambda_\mathrm{a} = 4$) leads to more pronounced apical indentation of folds arising from the decrease of basal surface and line tensions or increase in lateral surface tension.

Note that we restrict ourselves here to folds forming in columnar cells, with surface and line tensions changing in a narrow stripe of ~ 4 cells. It would be interesting to explore whether changes in forces occurring in larger stripes or in more cuboidal cells lead to different deformations than observed here.

Discussion of analysis of laser ablation experiments

Here we discuss briefly the analysis of recoil following a laser ablation experiment. We denote l the distance between two vertices around an ablated junction, and l_0 the initial distance before the laser ablation ($l(t = 0) = l_0$). Describing the relaxation of the junction by a Kelvin-Voigt model [3–6], we have

$$\mu \frac{dl}{dt} + k(l - l_0) = \Lambda \tag{27}$$

where Λ is the line tension of the junction, and μ, k are the effective viscosity and effective elasticity in the Kelvin-Voigt description. For a constant line tension Λ, Supplementary Equation 27 can be solved to yield the overall displacement:

$$\epsilon = l - l_0 = \frac{\Lambda}{k}\left(1 - e^{-\frac{kt}{\mu}}\right) \quad . \tag{28}$$

Therefore, if the parameters k and μ are constant, the displacement at a given time t is proportional to the line tension Λ, with a proportionality constant $\left(1 - e^{-\frac{kt}{\mu}}\right)/k$. For two junctions 1 and 2 with different line tensions Λ_1 and Λ_2 and same viscosities and elasticity μ and k, and defining the average velocity of junction i $v_i(t) = \epsilon_i(t)/t$, one can write

$$\frac{v_1(t)}{v_2(t)} = \frac{\Lambda_1}{\Lambda_2} \tag{29}$$

so that average recoil velocities measured at a fixed time point can be used to compare line tensions. Here we use average velocities measured at a fixed time $t = 250$ms for apical and basal cuts and $t = 1$s for cuts of lateral junctions. In both cases, the earliest time point after ablation at our time resolution was chosen, in order to avoid possible cytoskeletal rearrangements affecting the dynamics of recoil on slower time scales.

Supplementary References

1. Bielmeier, C. *et al.* Interface contractility between differently fated cells drives cell elimination and cyst formation. *Current Biology* **26,** 563–574 (2016).

2. Press, W. H. *Numerical recipes 3rd edition: The art of scientific computing* (Cambridge University Press, 2007).

3. Fernandez-Gonzalez, R., de Matos Simoes, S., Röper, J.-C., Eaton, S. & Zallen, J. A. Myosin II dynamics are regulated by tension in intercalating cells. *Developmental cell* **17,** 736–743 (2009).

4. Mayer, M., Depken, M., Bois, J. S., Jülicher, F. & Grill, S. W. Anisotropies in cortical tension reveal the physical basis of polarizing cortical flows. *Nature* **467,** 617 (2010).

5. Bonnet, I. *et al.* Mechanical state, material properties and continuous description of an epithelial tissue. *Journal of The Royal Society Interface* **9,** 2614–2623 (2012).

6. Fischer, S. C. *et al.* Contractile and mechanical properties of epithelia with perturbed actomyosin dynamics. *PLoS One* **9,** e95695 (2014).
