# Supplementary material for: Differential lateral and basal tension drive folding of Drosophila wing discs through two distinct mechanisms
Source: Nat Commun. 2018 Nov 5;9:4620. doi: 10.1038/s41467-018-06497-3 (PMC6218478; doi:10.1038/s41467-018-06497-3)
Supplement: Supplementary file 3 — Description of Additional Supplementary Files [file 41467_2018_6497_MOESM3_ESM.docx]

**Description of Additional Supplementary Files**

File Name: Supplementary Movie 1

Description: Dynamics of fold formation A time-lapse movie showing apical (above) and cross-sectional (below) views of a cultured wing imaginal disc expressing Indy-GFP to mark cell membranes. Times relative to the onset of apical invagination of the H/H fold are indicated. Scale bars are 10μm.

File Name: Supplementary Movie 2

Description: Tracking the shapes of RFP-marked cells during folding Time-lapse movies showing top (above) and cross sectional (below) views of cultured wing imaginal discs expressing Indy-GFP in all cells (grey) and RFP in clones of cells (red). Movies on the left and right show the formation of the H/H and H/P folds, respectively. Lines indicate position of cross-sectional views. Scale bars are 10μm.

File Name: Supplementary Movie 3

Description: Cell proliferation is not required for epithelial folding Time-lapse movies showing apical (above) and cross-sectional (below) views of control and *Cdk1^E1-24^* mutant cultured wing imaginal discs expressing E-cad-GFP. Time after shift to the restrictive temperature is shown. Green and magenta arrows mark H/H and H/P fold, respectively. Scale bars are 10 μm.

File Name: Supplementary Movie 4

Description: Tissue relaxation is increased for ablation of basal cell edges compared to apical cell edges Time-lapse movies showing wing imaginal disc pouch cells of 72h AEL larvae expressing IndyGFP before and after ablation of a single cell edge at the apical (left) or basal (right) side of the epithelium. Red dots mark vertices of ablated cell edges. Time is indicated in seconds after the start of the movie and the laser ablation occurs at around 14s. Scale bars are 10μm.

File Name: Supplementary Movie 5

Description: Collagen is required for proper wing imaginal disc shape and folding Time-lapse movie showing apical (above) and cross-sectional (below) views of a 76h AEL cultured wing imaginal disc co-expressing Indy-GFP and Vkg-GFP (to mark Collagen IV) after 9 addition of collagenase to the medium. Green and magenta arrows mark H/H and H/P fold, respectively. Scale bars are 10 μm.

File Name: Supplementary Movie 6

Description: Dynamics of F-actin and cell shape in H/P fold cells (a) Time-lapse movie showing a cross-sectional view of H/P fold cells expressing Utr-GFP to reveal the dynamics of F-actin and cell shape changes. (b) Time-lapse movie showing a XY view approximately 13μm below the apical cell surface and a cross-sectional view of H/P fold cells expressing Utr-GFP. (c) Time-lapse movie showing a XY view approximately 13 μm below the apical cell surface and a cross-sectional view of H/H fold (left) and H/P fold (right) cells expressing Utr-GFP. Scale bars are 10 μm.

File Name: Supplementary Movie 7

Description: Dynamics of apical F-actin and apical cell shape change in H/P fold cells Time-lapse movie showing apical and cross-sectional views of H/P fold cells expressing UtrGFP to visualize the dynamics of F-actin and cell shape changes. Magenta arrow marks H/P fold. Scale bars are 10 μm.

File Name: Supplementary Movie 8

Description: Dynamics of basal F-actin and basal cell shape change in H/P fold cells Time-lapse movie showing basal and cross-sectional views of H/P fold cells expressing UtrGFP to visualize the dynamics of F-actin and cell shape changes. Magenta arrow marks H/P fold. Scale bars are 10 μm.

File Name: Supplementary Movie 9

Description: Lateral cell interfaces accumulating F-actin are under high mechanical tension Time-lapse movies showing cross-sectional views of neighboring (left) or H/P fold (right) cells expressing Utr-GFP before and after ablation of single lateral cell interfaces. The lateral cell interfaces of the H/P fold cell accumulate F-actin shortly before laser ablation. Red bars mark time and approximate location of laser ablation. Note that the time delay changes from an initial 10 sec. to 1 sec. Scale bars are 10μm. 10

File Name: Supplementary Movie 10

Description: Three-dimensional vertex model simulations of epithelial folding by apical constriction, basal relaxation and lateral constriction Movies showing the apical (upper panel) and basal (lower panel) view of the epithelial geometry for successive minimization steps of an initially equilibrated homogeneous tissue, where the mechanical properties in a stripe of cells are changed according to the three mechanisms. Note that the sequence of minimization steps does not represent a realistic dynamic representation of the presented processes, since dissipative processes are not taken into account in simulations shown here. (a,b) Apical constriction simulations. The apical surfaces of the pre-fold cells are increased by a factor 5 compared to surrounding cells. (c,d) Basal relaxation simulations. The basal tension of pre-fold cells is decreased by 60% compared to non-folding cells. (e,f) Lateral constriction simulations. The lateral surface tension between prefold cells is increased by a factor 3 compared to non-folding cells.
